# Using Health and Well-Being Apps for Behavior Change: A Systematic Search and Rating of Apps

**DOI:** 10.2196/11926

**Published:** 2019-07-04

**Authors:** Fiona H McKay, Annemarie Wright, Jane Shill, Hugh Stephens, Mary Uccellini

**Affiliations:** 1 Deakin University School of Health and Social Development Burwood Australia; 2 Victorian Health Promotion Foundation (VicHealth) Carlton Australia; 3 The University of Melbourne (Honorary) Parkville Australia; 4 Dialogue Consulting Melbourne Australia

**Keywords:** smartphone, mobile apps, health promotion, health behavior, rating

## Abstract

**Background:**

Smartphones have allowed for the development and use of apps. There is now a proliferation of mobile health interventions for physical activity, healthy eating, smoking and alcohol cessation or reduction, and improved mental well-being. However, the strength or potential of these apps to lead to behavior change remains uncertain.

**Objective:**

The aim of this study was to review a large sample of healthy lifestyle apps at a single point in time (June to July 2018) to determine their potential for promoting health-related behavior change with a view to sharing this information with the public. In addition, the study sought to test a wide range of apps using a new scale, the App Behavior Change Scale (ABACUS).

**Methods:**

Apps focusing on 5 major modifiable lifestyle behaviors were identified using a priori key search terms across the Australian Apple iTunes and Google Play stores. Lifestyle behavior categories were selected for their impact on health and included smoking, alcohol use, physical activity, nutrition, and mental well-being. Apps were included if they had an average user rating between 3 and 5, if they were updated in the last 18 months, if the description of the app included 2 of 4 behavior change features, and if they were in English. The selected behavior change apps were rated in 2 ways using previously developed rating scales: the Mobile App Rating Scale (MARS) for functionality and the ABACUS for potential to encourage behavior change.

**Results:**

The initial search identified 212,352 apps. After applying the filtering criteria, 5018 apps remained. Of these, 344 were classified as behavior change apps and were reviewed and rated. Apps were given an average MARS score of 2.93 out of 5 (SD 0.58, range 1.42-4.16), indicating low-to-moderate functionality. Scores for the ABACUS ranged from 1 to 17, out of 21, with an average score of 7.8 (SD 2.8), indicating a low-to-moderate number of behavior change techniques included in apps. The ability of an app to encourage practice or rehearsal, in addition to daily activities, was the most commonly identified feature across all apps (310/344, 90.1%), whereas the second most common feature was the ability of the user to easily self-monitor behavior (289/344, 84.0%).

**Conclusions:**

The wide variety of apps included in this 2018 study and the limited number of behavior change techniques found in many apps suggest an opportunity for improvement in app design that will promote sustained and significant lifestyle behavior change and, therefore, better health. The use of the 2 scales for the review and rating of the apps was successful and provided a method that could be replicated and tested in other behavior change areas.

## Introduction

### Modifiable Risk Factors for Chronic Disease

Life expectancies across many developed countries have steadily increased over the past century. Newborns in developed countries can expect to live for 80 years or more, with an average increase of 3 years per decade [1]. At the same time, death rates in developed countries continue to fall, with the leading underlying causes of death being age-related diseases, including coronary heart and Alzheimer disease and cancers [1]. Although these overall patterns are encouraging and suggest that activities to improve health are working, areas of concern remain. An analysis of data reporting on the global burden of disease suggests that although overall life expectancy has increased, much of the burden of disease could be prevented through a reduction in exposure to modifiable risk factors, including tobacco use, high body mass, high alcohol use, physical inactivity, and high blood pressure [2]. Rates of overweight and obesity have increased, with the vast majority of adults and children not consuming the recommended quantities of fruits and vegetables or engaging in the recommended amount of daily physical activity [2]. Although rates of smoking, daily alcohol use, and overall alcohol consumption have decreased somewhat in the adult population in recent years, they remain significant contributors to disease burden [2]. In addition to the focus on physical health, global data related to mental health indicate that approximately 1 in 4 people worldwide will experience a diagnosable mental illness over their lifetime [2].

### Interventions to Address Modifiable Risk Factors

A large amount of research has investigated the variety of responses to the most common modifiable risk factors: obesity, physical inactivity, poor nutrition, tobacco use, risky alcohol use, and poor mental health. In the quest for solutions for poor health, this research has explored the clinical as well as community setting, proposing solutions that exist at the societal, environmental, household, and individual level. Much of the research investigating ways to increase physical activity is focused on children and adolescents, as most adolescents are physically inactive, with this inactivity continuing into adulthood [3].

A recent review found that, similar to many high-income countries, chronic disease is Australia’s biggest health challenge, responsible for 83% of premature deaths [4]. This review found that interventions targeting the workplace, facilitated group-based exercise programs, the promotion of activities that encourage or provide the ability to self-monitor behavior, tobacco cessation programs that embed coaching or counselling, and practical support for weight loss are most likely to be effective [4]. Although interventions that have little effectiveness include those focused on education or awareness raising, particularly around healthy food and beverage options, modifications to workplace and community environments have been identified as a way to encourage physical activity as have interventions that include financial rewards or penalties [4].

### Smartphone Apps for Health

Although this evidence points to some success in supporting positive health by modifying risk factors, the intensive nature and expense often associated with these programs, can prohibit the large-scale rollout of such interventions. Smartphone apps represent a potential supplement to these efforts that could lead to substantial population-level impact and long-term health behavior change. Approximately 70% of the populations of developed countries own a smartphone [5]. The versatility of smartphones has led to the creation of millions of apps beyond those originally supplied to consumers, such as mail, map, and messaging apps, and now include gaming, banking, recipe finders, and health apps. Apps are created by developers and can be downloaded from a variety of digital marketplaces depending on the operating system of the device. Such technologies make available interventions that are low cost and can be accessed by much of the population.

In recent years, there has been a proliferation of apps designed to improve health. This has included apps that promote physical fitness through attendance at gyms or via counting steps, apps that track calorie intake and suggest modifications, apps that aim to assist users with smoking cessation or reducing alcohol consumption, and apps that promote mindfulness and positive mental health. However, it is unclear if these apps follow best practices in app design or health behavior change, or indeed what is the best practice for designing health behavior change apps. Although some studies have reported on the behavior change content of apps, for example, smoking cessation [6], alcohol reduction [7,8], physical activity [9], or for specific medical conditions [10], there is also research suggesting that many apps fail to include techniques or features that have been shown to be effective in behavior change, such as the ability to be customizable to users’ needs or personal characteristics or to be responsive to change [7,10,11]. There is also a risk that improper or unsupervised use of apps or the use of apps that do not align with current recommendations may result in harmful outcomes for users [12,13].

### Evaluation of Mobile Health Apps

Part of the problem in evaluating the effectiveness or accuracy of information in apps is related to limitations in the methods available and inconsistencies in the approach to research in this area. Using smartphone apps for healthy behavior change is an emerging area of investigation, and as a result, much of the current research is focused on the evaluation of single apps [14] and apps that have been purposely designed for a research project [6], or a small number of *top-rated apps* [15,16], rather than focusing on a thorough large-scale investigation of the potential of apps that already exist on the market to promote behavior change [17].

A recent systematic review investigated approaches to the evaluation of health apps with the aim of identifying current best practice approaches [18]. The review of 38 papers found no single best practice method of evaluating mobile health and well-being apps. Most approaches did not include sufficient information or evaluation, potentially meaning consumers are provided with incomplete and inaccurate information about the apps. The review suggested that the evaluation of apps should include a review of the functionality and usability of the app, as well as an assessment of the apps’ potential to promote behavior change. It found that although not specific for smartphone apps, the Coventry, Aberdeen, and London—Refined taxonomy, developed by Michie et al [19], was the most commonly used instrument for assessing behavior change techniques in interventions and the Mobile Apps Rating Scale (MARS), developed by Stoyanov et al [20] was the most commonly used tool for assessing the quality and functionality of mobile health apps.

On the basis of this review, McKay et al [21] developed a scale specifically designed to determine the behavior change potential of smartphone apps. This tool, the App Behavior Change Scale (ABACUS), based off the health behavior change intervention literature, is a 21-item instrument that reports high percentage agreement, Krippendorff alpha, interrater reliability, and high internal consistency.

This study aims to rate the potential effectiveness of the apps using a scale that assesses the inclusion of features that are known to assist individuals with behavior change, designed to reduce alcohol consumption or smoking, improve nutrition or mental well-being, or increase physical activity to provide potential app users information on the content of apps and their likely effectiveness in supporting the user’s lifestyle behavior change goals.

The ratings are housed on a website developed by the Victorian Health Promotion Foundation (VicHealth) to assist the public in making informed choices about effective healthy lifestyle apps. VicHealth is a public health agency based in the Australian state of Victoria that is focused on promoting good health and preventing chronic disease. The previously mentioned 5 healthy lifestyle areas were selected as these were the focus of VicHealth’s programs and reflected major lifestyle risk factors that contributed to the burden of disease in Australia [22].

In addition, this study also sought to describe the method used in the rating of apps for health-related behavior change with a view to enabling application of the method to regular review and public update about the effectiveness of health apps. This approach has a broad application in other health areas where the assessment of an app’s potential to support effective adoption of healthy behaviors is required and where health agencies or government health departments have a mandate to support the public in making effective choices for health. This is particularly critical for app consumers as there is a proliferation of health apps on the market with claims regarding their effectiveness in supporting health behaviors, including behaviors related to a healthy lifestyle and illness self-management. Application of a consistent app review method can simplify the process of regularly reviewing apps on the market to keep the public informed of their potential effectiveness.

## Methods

This study employed 2 scales to rate smartphone apps. The first was MARS [20] for functionality, and the second was ABACUS [21] to determine the potential for behavior change. Independent raters applied these scales to the apps identified from the Apple iTunes and Google Play stores.

### Sample Selection

**Figure 1:**
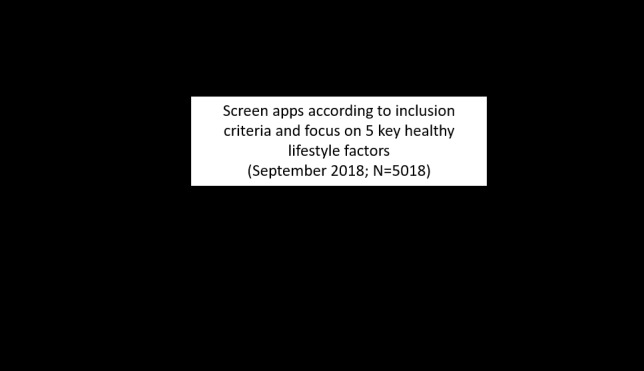
Process for identifying and rating apps.

The Australian Apple iTunes and Google Play stores were searched to identify health and well-being apps using a priori key search terms outlined below from June to July 2018. Identified apps underwent a 4-step screening, review, and rating process (see Figure 1).

All apps available for download in Australia containing a predefined keyword in either the title or description were collected from the Google Play and Apple iTunes stores from June to July 2018. Search terms were developed by health promotion experts in the fields of alcohol and tobacco cessation, healthy eating, physical activity, and mental well-being. This study sought to investigate apps that would encourage health behavior change, rather than those that would simply enable health promotion; therefore, search terms that would identify a specific condition were not included. The search terms used were as follows: health, lifestyle, alcohol, alcoholic, drinks, drinking, booze, sober, blood alcohol, BAC, smoking, smoke, cigarette, tobacco, fitness, exercise, running, exercising, physical activity, active, steps, walking, training plan, nutrition, social isolation, healthy eating, diet, healthy eating, healthy food, health food, healthy drink, health drink, water, hydration, junk food, salt, sodium, social connection, anxiety, well-being, relaxation, mindfulness, stress, mood, meditate, meditation, emotional intelligence, empathy, gratitude, loneliness, friendship, resilience, and resilient. Apps were filtered according to the following inclusion criteria: average user rating of greater than or equal to 3 and at least 10 user reviews (all versions), updated in the last 18 months, and in the English language. Apps were then categorized into 5 key healthy living categories: promoting healthy eating, encouraging regular physical activity, preventing tobacco use, reducing alcohol consumption, and improving mental well-being.

Apps were further filtered and excluded if conflicts of interest were identified, for example, apps promoting negative behavior such as prosmoking in tobacco apps; the app targeted a specific clinical population such as people living with diabetes, or the treatment of psychological disorders, as this review sought to evaluate apps that encourage health behavior change through health promotion and not clinical management of a disease or condition; or the app was not relevant to Australia, including those where a currency other than Australian dollars was only used, nonmetric measures, or a gym franchise in countries other than Australia.

Following this, app descriptions were reviewed independently by 2 reviewers to determine if they could be classified as promoting behavior change. Behavior change, in this instance, refers to the new activities or actions one needs to do regularly to achieve a healthier lifestyle, for example, exercising, eating healthier food, or managing stress. To investigate the potential for behavior change, app descriptions were reviewed against 4 criteria: (1) the ability to set goals for the actions the user would like to achieve, (2) the ability to tailor the app, (3) the ability to share progress with others (for example, through connections on social media), and (4) the ability to receive rewards or acknowledgments when activities are completed or progress toward a goal is made. These criteria are based on previous health promotion and behavior change research [23]. As this review was interested in apps that had the potential to change behavior, apps that only provided information or connected users to a service or facility and did not meet at least 2 of the 4 behavior change elements listed above were excluded.

All behavior change apps were downloaded for use on an iPhone. If an app was not available for one of those platforms (or was incompatible with the device used), reviewers identified and used a compatible device. Where an app was available on both Google Play and Apple iTunes, for ease, the apps were downloaded for review on an Apple device. When data were collected, the description for apps on Google Play and Apple iTunes were compared. Similarities and differences in app description were noted at this point; however, if the app was described the same way (excluding differences in terms of the user experience, design, or layout as these are expected across the different platforms), the app was considered identical across both Android and Apple platforms. However, this review did not validate every feature of every app across both platforms, as such, there is a possibility that some apps may have differences when used on an Android versus iOS device.

### Functionality Review and Rating

The functionality rating uses the MARS [20]. This rating scale was used to examine app elements, such as engagement, functionality, utility, aesthetics, and information. This scale includes 23 items across 5 categories with each item scored using a series of questions on a 5-point ordinal scale response. An overall functionality score out of 5 was derived using this scale.

All apps were scored by at least 2 people with expertise in reviewing apps. To standardize the approach between reviewers, a pilot of a small group of apps was initially conducted and results were compared. Each app was downloaded and, consistent with other studies [15,20], used for approximately 10 min to allow the rater to familiarize themselves with the functionality of the app and user experience. Reviewers attempted to use all parts of each app, noting if the app crashed or its functions were not accessible. Where functionality scores differed, the reviewers considered the app together, sought consensus, and determined a final score.

Technical features of apps were also recorded but did not form part of the functionality rating. These features included the following: whether the app had a privacy policy, required login, allowed password protection, allowed for social media integration, allowed data export, had an app community, sent reminders, needed Web access to function, required add-ons such as a fitness band to use the app (and whether this was a one-off purchase, such as a fitness tracker or an ongoing purchase), the presence of in-app-advertising or payments (product purchases made within the app, such as unlocking extra features including videos or removing advertisements), and whether the app asked permissions to send push notifications.

### Behavior Change Potential Review and Rating

The ABACUS [21] was used to measure behavior change potential. This rating scale comprises 21 items and was used to examine the potential behavior change of the app in relation to goal setting, action planning, barrier identification, self-monitoring, and feedback.

All apps were reviewed and scored by at least 2 reviewers with expertise in public health and health promotion. Interrater reliability was examined with good reliability observed between reviewers (intraclass correlation .906 [95% CI 0.854-0.936]). Each app was first explored by the reviewer to gain familiarity with the app and the interface. The reviewers used all app functions including images, cartoons, videos, record keeping, calendars, and reminders. A total theoretical score out of 21 was calculated by summing the item scores.

### Data Analysis

Basic descriptive statistics were conducted to characterize the sample. Percentages are presented for categorical variables and means, or for medians presented for continuous variables. All analyses were conducted using SPSS for Windows, version 22.0 (SPSS Inc).

## Results

The initial search was conducted between June 28 and July 2, 2018 and identified 212,352 apps. After applying the filtering criteria, 5018 apps remained. Of these, 356 were classified as behavior change apps, of which 12 were unavailable at the time of review and eventually removed (11 physical activity apps and 1 mental well-being app), leaving 344 for review. These apps were categorized as either physical activity (n=275), healthy eating (n=23), mental well-being (n=27), tobacco (n=14), or alcohol (n=5). All apps were reviewed by 2 reviewers.

Most apps were free (279/344, 81.1%), and all were available on the iTunes platform (344/344, 100.0%), with over two-thirds (236/344, 68.6%) also available on the Google Play store. Around half of the apps required some form of purchase, for example, 171 apps (171/344, 49.7%) required an ongoing purchase including membership or subscription, whereas 141 apps (141/344, 40.9%) required a one-off purchase. Physical activity apps were most likely to require some form of in-app payment (191/344, 55.5%) or one-off purchase (113/344, 32.8%). Two-thirds of all apps (233/344, 67.7%) required some form of in-app payment, typically allowing the user to unlock a feature or to remove advertisements. Advertisements were identified in 93 apps (93/344, 27.1%), mostly in those that were categorized as physical activity apps (75/344, 21.8%); see Table 1 for more app features.

All apps were assessed against both the MARS [20] and ABACUS [21] (see Multimedia Appendix 1 for an overview of scores). Using the MARS, apps were assigned a score out of 5. Apps in this review were given an average score of 2.93 (SD 0.58, range 1.42-4.16), indicating moderate functionality across all apps. Table 2 shows the average scores for the whole sample from the highest average score to the lowest on the MARS. Overall, accuracy of description (3.88), performance (3.30), and layout (3.42) were the highest rating features, whereas the credibility of the app (2.11) was the lowest scoring feature.

The apps that were categorized as mental well-being received the highest MARS scores (average of 3.26), and apps categorized as promoting healthy eating received the lowest MARS scores (average of 2.71). Apps that were categorized as improving mental well-being also scored the highest on many of the individual elements. For example, mental well-being apps scored highest in the elements of accuracy of design (4.17), performance (3.78), target group (3.75), gestural design (3.64), layout (3.6), graphics (3.53), quality (3.43) and quantity (3.05) of information, visual appeal (3.35), interest (2.67), credibility (2.64), visual information (2.5), and entertainment (2.28).

The ABACUS was applied to all apps, resulting in a score for each app from 0 to 21. Scores in this review ranged from 1 to 17, with an average score of 7.8 (SD 2.8), indicating a low-to-moderate number of behavior change techniques included in apps. The apps categorized as tobacco cessation scored the highest on the ABACUS indicating the highest number of behavior change features, with an average of 10.2 in each of the 14 apps rated; this was followed closely by apps categorized as improving mental well-being and promoting healthy eating, which were identified as having on average 8.7 and 8.6 items, respectively. The ability of an app to encourage practice or rehearsal in addition to daily activities was the most commonly identified feature of all apps in total (310/344, 90.1%), and specifically the apps categorized as increasing physical activity (263/275, 95.6%), healthy eating (20/23, 86%), and improving mental well-being (23/27, 85.2%). The second most common feature across all apps was the ability of the app to allow the user to easily self-monitor behavior. This feature was identified in 84.0% (289/344) of apps across all categories and in 100% (14/14) of smoking cessation apps. Apps aiming to reduce alcohol consumption were identified as having the fewest features that could promote behavior change; however, this finding needs to be interpreted with caution as there were only a small number of apps (n=5) in this category (see Table 3 for more details on the frequency of each behavior change technique).

**Table 1:** App features.

Features	Total sample (n=344)	Increasing physical activity (n=275)	Promoting healthy eating (n=23)	Improving mental well-being (n=27)	Preventing tobacco use (n=14)	Reducing alcohol consumption (n=5)
Price (Aus $), mean (SD)	1.19 (3.73)	1.27 (3.8)	0.78 (1.74)	0.89 (3.00)	0.89 (2.9)	0.89 (2.01)
User rating, mean (SD)	4.43 (0.41)	4.44 (0.39)	4.32 (0.36)	4.5 (0.54)	4.28 (0.47)	4.6 (0.42)
Platform availability—Apple iTunes, n (%)	344 (100)	275 (100)	23 (100)	27 (100)	14 (100)	5 (100)
Can be used without add-ons, n (%)	194 (56.4)	182 (66.2)	22 (95)	27 (88)	14 (100)	5 (100)
Requires in-app payments, n (%)	233 (67.7)	191 (69.5)	15 (65)	17 (62)	8 (57)	2 (40)
One-off purchase required, n (%)	141 (40.1)	113 (41.1)	10 (43)	10 (37)	6 (42)	2 (40)
Had in-app advertisements, n (%)	93 (27.1)	75 (27.2)	8 (34)	3 (11)	6 (42)	1 (20)
Had a privacy statement, n (%)	344 (100)	275 (100)	23 (100)	27 (100)	14 (100)	5 (100)
Allowed password protections, n (%)	32 (9.3)	25 (9.0)	3 (13)	4 (14)	0 (0)	0 (0)
Allowed data to be exported, n (%)	36 (13.4)	35 (12.7)	6 (26)	5 (18)	0 (0)	0 (0)
Allowed sharing, n (%)	219 (63.7)	191 (69.5)	8 (34)	11 (40)	10 (71)	0 (0)
Had an app community, n (%)	106 (30.8)	88 (32.0)	3 (13)	6 (22)	9 (46)	1 (20)
Required login, n (%)	134 (38.9)	110 (40.0)	8 (34)	14 (51)	2 (14)	1 (20)
Sent reminders, n (%)	215 (62.5)	168 (61.1)	14 (60)	20 (74)	12 (85)	2 (40)
Needed Web access to function, n (%)	118 (34.3)	94 (34.2)	6 (26)	12 (44)	5 (35)	1 (20)
Asked permission for push notifications, n (%)	225 (65.4)	182 (66.2)	14 (60)	17 (62)	11 (78)	1 (20)

**Table 2:** Performance on individual Mobile App Rating Scale elements (highest to lowest).

Item	Mean score	Increasing physical activity (score)	Promoting healthy eating (score)	Improving mental well-being (score)	Preventing tobacco use (score)	Reducing alcohol consumption (score)
Accuracy of description (in app store)	3.88	3.82	3.77	4.17	4.06	4.09
Gestural design	3.30	3.28	3.18	3.64	3.2	3.45
Performance	3.49	3.45	3.32	3.78	3.46	4.0
Target groups	3.33	3.33	3.09	3.75	3.06	3.36
Ease of use	3.29	3.29	3.00	3.60	3.00	3.63
Navigation	3.20	3.19	3.14	3.32	3.13	3.45
Layout	3.42	3.46	3.05	3.60	3.13	3.18
Graphics	3.20	3.20	3.0	3.53	2.8	3.27
Visual appeal	2.94	2.96	2.68	3.35	2.6	2.36
Customization	2.88	2.92	3.05	2.82	2.8	1.81
Interactivity	3.23	3.23	3.36	3.28	3.53	2.54
Interest	2.30	2.29	2.0	2.67	2.06	2.63
Entertainment	2.16	2.19	2.09	2.28	1.93	1.54
Visual information	2.31	2.39	1.59	2.5	2.0	1.81
Goals	2.97	2.88	3.18	3.25	3.6	3.0
Credibility	2.11	2.03	1.91	2.64	2.46	2.63
Quality of information	2.49	2.38	1.77	3.43	2.73	3.9
Quantity of information	2.18	2.11	1.64	3.05	2.2	2.9
Overall rating	2.93	2.93	2.71	3.26	2.75	2.82

**Table 3:** Performance on App Behavior Change Scale (ABACUS) criteria (most to least frequently used).

Behavior change technique	Frequency	Increasing physical activity	Promoting healthy eating	Improving mental well-being	Preventing tobacco use	Reducing alcohol consumption
Allow or encourage practice or rehearsal in addition to daily activities, n (%)	310 (90.1)	263 (95.6)	20 (86.5)	23 (85.2)	2 (14.2)	2 (40)
Allow the user to easily self-monitor behavior, n (%)	289 (84.0)	238 (86.5)	19 (82.5)	17 (63.0)	14 (100)	1 (20)
Provide instruction on how to perform the behavior, n (%)	233 (67.7)	197 (71.6)	7 (30.5)	23 (85.2)	3 (21.4)	3 (60)
Customize and personalize some features, n (%)	227 (60.0)	186 (67.6)	10 (43.5)	18 (66.7)	10 (71.4)	3 (60)
Reminders and/or prompts or cues for activity, n (%)	225 (65.4)	184 (66.9)	14 (60.5)	18 (66.7)	8 (57.1)	1 (20)
Baseline information, n (%)	192 (55.8)	154 (56.0)	15 (65.5)	6 (22.2)	14 (100)	3 (60)
Give user feedback (person or automatic), n (%)	191 (55.5)	164 (59.6)	4 (17.5)	20 (74.1)	2 (14.2)	1 (20)
Encourage positive habit formation, n (%)	186 (54.1)	151 (54.9)	12 (52.5)	19 (70.4)	2 (14.2)	2 (40)
Share behaviors with others and/or allow for social comparison, n (%)	155 (45.1)	129 (46.9)	4 (17.5)	10 (37.0)	11 (78.5)	1 (20)
Provide general encouragement, n (%)	138 (40.1)	105 (38.1)	8 (34.5)	15 (55.6)	9 (64.2)	1 (20)
Goal setting, n (%)	102 (29.7)	69 (25.0)	20 (86.5)	4 (14.8)	6 (42.8)	3 (60)
Review goals, update, and change, n (%)	98 (28.5)	65 (23.6)	20 (86.5)	3 (11.1)	7 (50)	3 (60)
Understand the difference between current action and future goals, n (%)	83 (24.1)	51 (18.5)	15 (65.5)	2 (7.4)	13 (92.8)	2 (40)
Material or social reward or incentive, n (%)	69 (20.1)	53 (19.2)	7 (30.5)	3 (11.1)	5 (35.7)	1 (20)
App created with expertise and/or information consistent with national guidelines, n (%)	60 (17.4)	27 (9.81)	12 (52.5)	10 (37.0)	9 (64.2)	2 (40)
Information provided about the consequences of continuing and/or discontinuing behavior, n (%)	34 (9.9)	11 (4.0)	1 (4.5)	6 (22.2)	13 (92.8)	3 (60)
Restructure the physical or social environment, n (%)	30 (8.7)	5 (1.81)	3 (13.5)	17 (63.0)	3 (21.4)	2 (40)
Distraction or avoidance	23 (6.7)	3 (1.09)	2 (8.5)	12 (44.4)	4 (28.5)	2 (40)
Provide the opportunity to plan for barriers, n (%)	22 (6.4)	4 (1.45)	4 (17.5)	9 (33.3)	3 (21.4)	2 (40)
Data export, n (%)	18 (5.2)	15 (5.45)	0 (0)	1 (3.7)	2 (14.2)	0 (0)
Willingness for behavior change, n (%)	12 (3.5)	7 (2.54)	0 (0)	0 (0.0)	3 (21.4)	2 (40)
ABACUS average score	7.8	7.56	8.6	8.7	10.2	8.0

## Discussion

### Principal Findings

This study shows the extent to which smartphone apps incorporate behavior change techniques and basic functionality by using 2 validated scales, the MARS [20] and ABACUS [21]. This study extends on previous app review work by examining a greater number of apps across 5 separate categories and testing the ABACUS for the first time, on a large number of apps [21]. Despite the increase in sample, the results for this Australian study are consistent with past research investigating the inclusion of behavior change techniques in apps for alcohol [24] and smoking cessation [25], weight loss [26,27], and improved physical activity [28], showing that smartphone apps include a limited number of behavior change techniques.

The most common behavior change techniques included in apps in this study were those related to practice and rehearsal, instruction, self-monitoring behavior, customizing features, and the inclusion of reminders or prompts of activity. Given the large amount of research identifying goal setting as important in achieving behavior change [29,30] and with goal setting shown to increase success in changing behaviors around nutrition [31] and physical activity [32], it is disappointing to note that only around one-third of apps included an option for users to set and change goals, with many of the apps only allowing for the review of automatic goals. It is also interesting to note that the ability to plan for barriers, export data from the app (for example, to a health care professional), or gather background on willingness for behavior change were not prominent in the apps reviewed, despite the technology being readily available for these features and other research highlighting such features as important in encouraging positive behavior change [33].

Although we identified a very large number of physical activity apps (almost 80% of the sample) leading to a high level of consumer choice, we found that most included a limited number of techniques known to promote sustained behavior change. The apps that were categorized as promoting physical activity had on average 7 to 9 behavior change techniques. In over 90% of physical activity apps, the app allowed repeat practice or rehearsal. This feature allows users of the apps to engage in the behavior more than once each day, for example, in a yoga app, the user would be permitted to undertake more than 1 yoga session in a defined period. This is particularly important as behavior change intervention research suggests that repetition is an important component of a successful intervention [34].

Around one-quarter of the physical activity apps contained features allowing users to set their own goals or update these goals. Given the large number of physical activity apps on the market, this is a clear gap in app design, particularly given the large amount of research that has already been conducted on the importance of these features [23,35,36]. However, this is consistent with previous research which indicates that many of the physical activity apps on the market have limited behavior change features [37], and their features consist mainly of step-by-step instructions for particular exercises [35]. It is also disappointing to note that goal setting was missing from alcohol and tobacco cessation apps, given the importance of goal setting in decreasing negative health behaviors [38,39].

Apps that were categorized as improving mental well-being, promoting healthy eating, or reducing alcohol consumption were identified as having around 8 behavior change techniques. Although the small number of alcohol reduction apps received ratings across a large range (range 2-17), the mental well-being and healthy eating apps had ratings across a smaller range (4-16 and 1-12, respectively). Unlike physical activity apps, the healthy eating apps were more likely to include a function to set and revise goals, whereas the mental well-being apps were more likely to encourage positive habit formation, with many of the apps encouraging and enabling daily practice. Mental well-being apps were also identified as allowing users to monitor their behavior against a set goal. This is positive, as there is some emerging research on mental well-being apps showing that those apps with some self-monitoring features are more likely to have positive impacts [28,40].

### Limitations

Although there are some interesting findings presented here, there are limitations to this study. First, this study features apps that were available in the Australian Apple iTunes and Google Play stores, and we found no apps that were present in the Google Play store only. Apps that did not allow Australian currency and that did not service an Australian audience were excluded. This could mean that we have missed apps that promote behavior change but are specific to another market. As such, caution needs to be taken when extrapolating these findings to other countries.

It may also be possible that by downloading apps from the Apple iTunes store that were also available in the Google Play store, we have missed an app that has an identical name and description in both stores but different features. Although the listings for apps were compared as part of the identification process to ensure that they were similar, we did not review or rate duplicate apps, and as such, there is a chance that some apps may have differences when used on an Android versus iOS device.

It is important to note that this study only presents an analysis of apps available at a single point in time: 2018. Although this research provides a reference point for further research into the quality of apps and a review of a large number of apps, given the fast-moving nature of this field, some of these apps may no longer be available or may have had their features updated.

A further limitation is the absence of content assessment of health information included within the apps. Although there has been some content investigation of single apps, or a small set of apps, this study is still in its infancy and represents an area for future investigation. Within this review, we also did not seek to measure the quality of the advice given or the relationship between the information provided with that of national and international guidelines.

Finally, with these data, we are unable to draw any conclusion relating to long-term behavior change. Given that we know that sustaining long-term behavior change is difficult, that even those programs that are effective have been shown to result in small changes [41], and that apps are typically used only on a few occasions before they are deleted [24,42], this is an area that needs more research attention as we strive to create apps that will be able to assist in improving the health of a large number of people at a low cost.

### Conclusions

The wide variety in apps and the low number of behavior change techniques found in most apps included in this 2018 study suggests an opportunity for growth in apps that can promote sustained and significant behavior change. Furthermore, the small number of apps on the market for reducing alcohol and tobacco consumption may also represent an opportunity for developers to create high quality apps that can assist with behavior change in these areas. Given that app development outpaces research and knowledge translation, it is difficult to see a time where apps will be based on best practice or most up-to-date behavior change techniques. However, with the increasing body of research identifying limitations in current apps, there is a potential for the creation of apps to more likely encourage behavior change. To this end, this research is complemented by a set of guidelines for app developers to assist them in developing apps that can effectively support lifestyle behavior change [43].

Overall, the use of the ABACUS [21] taxonomy for behavior change and the MARS [20] was successful. The reviewers reported having clear guidelines for the review, the time taken for each app was not prohibitive, and interrater reliability was good. Therefore, it provides a method that could be replicated and tested in other behavior change areas or used on a periodic basis to review apps available on the health app market to enable consumers to make optimal app selections.

## Appendix

Multimedia Appendix 1 Process for identifying and rating apps.

## Abbreviations

ABACUS: App Behavior Change Scale
MARS: Mobile App Rating Scale
VicHealth: Victorian Health Promotion Foundation
